# Feasibility of Using a Wearable Device to Identify Loneliness Among Older Adults Living Alone: Mixed Methods Study

**DOI:** 10.2196/94027

**Published:** 2026-10-15

**Authors:** Rajna Ogrin, Georgina Johnstone, Bradley R Crammond, Judy A Lowthian

**Affiliations:** 1 Bolton Clarke Research Institute, Bolton Clarke, Level 1, 347 Burwood Highway, Forest Hill, Victoria, 3131, Australia, 61 1300221122; 2 Monash Health, School of Clinical Sciences, Faculty of Medicine Nursing and Health Sciences, Monash University, Melbourne, Victoria, Australia; 3 Melbourne Medical School, Faculty of Medicine, Dentistry and Health Sciences, University of Melbourne, Melbourne, Australia; 4 Health Transformation Lab, RMIT University, Melbourne, Victoria, Australia; 5 School of Public Health and Preventive Medicine, Monash University, Melbourne, Victoria, Australia

**Keywords:** older adults, loneliness, usability, proof of concept, prototype, wearable electronic devices, design, co-design, mixed methods, digital health

## Abstract

**Background:**

Addressing loneliness is a public health priority, with increasing interest in technological solutions for older adults. However, evidence on real-world feasibility and acceptability of such technologies remains limited.

**Objective:**

This study evaluated the feasibility and acceptability of the Conversation as Therapy (CaT) Pin, a wearable device designed to capture time spent in conversation among older adults living alone, following prototype refinement. Specifically, the study assessed whether a sufficient amount of synchronized, device-derived conversation data and self-reported loneliness data could be generated to explore the relationship between conversation time and loneliness.

**Methods:**

A 2-phase, mixed methods feasibility study was conducted. In phase 1, a Consumer Advisory Group (CAG) participated in a 1-hour discussion to inform study processes and device design, followed by a 1-week prototype test with older adults (n=3). Prototype usability, device design, and study procedures were explored through interviews and informed iterative refinement. In phase 2, community-dwelling adults aged 65 years or older living alone trialed the device for 2 weeks. Participants completed activity logs capturing loneliness, activity engagement, device wear, and conversations at 6-hour intervals. Device-captured conversation time was compared with self-reported data where available. Qualitative interviews were analyzed using reflexive thematic analysis to understand participant experiences of device acceptability and feasibility.

**Results:**

In phase 1, 6 CAG participants, including 3 prototype testers, identified usability and design issues that led to modifications before phase 2. Phase 2 involved 23 participants (15 women; mean age 79.4 y, SD 6.7 y), with 7 classified as lonely using the UCLA-3 scale. However, only 8 (34.8%) participants generated sufficient concurrent device and activity log data, preventing meaningful assessment of the relationship between conversation time and loneliness. Feasibility challenges were driven by device acceptability, namely usability, comfort, aesthetics, and integration into daily life, which limited sustained wear and data synchronization. Qualitative findings indicated that participants prioritized quality rather than quantity of social interaction, and many did not perceive personal benefit from device use, citing lack of perceived need or concerns related to functionality and design. Together, methodological and conceptual challenges were identified in using conversation time as a potential indicator of loneliness.

**Conclusions:**

This study demonstrated that, in its current form, using a wearable device to capture time spent in conversation and align these data with self-reported loneliness was not feasible among older adults living alone. Feasibility was constrained by device acceptability related to usability, participant adherence, and data synchronization challenges. Findings also suggest that conversation time alone may be insufficient as an indicator of loneliness, given participants’ emphasis on the quality and personal meaning of social interaction. These results provide practical lessons for future digital health research with older adults, including the need for early co-design, monitorable data-capture systems, and more nuanced approaches to measuring loneliness.

## Introduction

With the increasing availability of digital health technologies, solutions are being developed to support older people in maintaining independence and promoting holistic well-being. However, older adults are heterogeneous, and technology uptake varies by factors including gender, education, and income [1]. Older end users remain underinvolved in technology development [1,2], which can result in products that are not fit for purpose, accessible [3], or appealing to intended users [4]. Ageism in digital design, whereby older people are implicitly perceived as having low competence or interest, contributes to this underinvolvement [5]. Therefore, despite rapid innovation, uptake by older community members remains low [6].

Loneliness is one priority area for technology-enabled approaches. Loneliness is defined as a subjective, unpleasant, or distressing feeling of a lack of connection to other people, along with a desire for more or more satisfying social relationships [7]. Social isolation is a distinct, separate domain, defined as having objectively fewer social relationships, social roles, group memberships, and infrequent social interaction [7]. Prolonged periods of social isolation and/or loneliness are associated with adverse outcomes, including poorer mental health (eg, social anxiety, clinical depression and suicidal ideation [8-10], dementia [11,12], cardiovascular disease [13], and increased mortality risk [14]). The COVID-19 pandemic further compounded these negative impacts, particularly among older community members [12,15].

Timely identification of loneliness is needed so that interventions can be implemented before loneliness becomes chronic. However, current approaches rely primarily on validated questionnaires [16], which may be affected by stigma and underreporting [17]. Emerging work has explored more time-sensitive approaches (eg, ecological momentary assessment via smart devices), though evidence remains limited in older adults living alone in real-world settings [18,19].

Social connection often involves conversation [20], with higher well-being associated with conversational patterns in daily life [21,22], and the quantity of social interactions associated with greater well-being [23] and feelings of belonging [24]. This has prompted interest in whether time spent in conversation could indicate potential loneliness. However, this raises methodological and conceptual challenges. Methodologically, passive detection requires sustained device wear, acceptable design, and robust data synchronization [25,26], while loneliness is subjective and relational, raising a conceptual tension between measuring conversation quantity and capturing the quality or meaning of social interaction.

In this context, we developed the Conversation as Therapy Pin, a prototype wearable device designed to estimate time spent in conversation while preserving privacy by not capturing conversational content. This 2-phase mixed methods feasibility study evaluated the acceptability and feasibility of using the CaT Pin with older adults living alone and whether sufficient synchronized device-derived conversation and self-reported loneliness data could be generated to explore the relationship between time spent in conversation and loneliness.

The research questions were as follows: in phase 1, what CaT Pin device usability or procedural issues arose during prototype testing and refinement? In phase 2, was the CaT Pin feasible and acceptable following phase 1 refinement, and could it generate sufficient synchronized, device-derived conversation and self-reported loneliness data to explore the relationship between time spent in conversation and loneliness in older adults living alone?

The study explored whether time spent in conversation could serve as a potential indicator of loneliness, contingent on generating sufficient synchronized data. We first describe the study procedures, then present feasibility and acceptability findings, including data completeness and synchronization, followed by qualitative findings on device experience and the conceptual appropriateness of conversation-based loneliness detection. We then conclude with implications for future digital health design and evaluation in older populations.

## Methods

### Study Design

This was a 2-phase prospective mixed methods feasibility study evaluating the acceptability, usability, and data capture feasibility of the CaT Pin for older adults living alone in the community. The primary feasibility question was whether sufficient synchronized, device-derived conversation and self-reported loneliness data could be generated to explore the relationship between time spent in conversation and loneliness.

Phase 1 involved prototype testing and procedural refinement. Phase 2 used a convergent mixed methods feasibility design, with quantitative and qualitative data collected concurrently and analyzed separately. The study was initially designed with a quantitative emphasis, with device-derived conversation data and repeated measures of loneliness used to explore the relationship between time in conversation and loneliness, while qualitative interviews would examine participant experiences and provide context for interpreting the quantitative findings. However, recruitment and data-capture challenges shifted the focus of the study toward feasibility and acceptability outcomes. A mixed methods approach was chosen because assessing feasibility required both quantitative and qualitative data, with combining methods providing a more comprehensive understanding than either approach alone.

This study is reported according to the GRAMMS (Good Reporting of a Mixed Methods Study) checklist for mixed methods studies [27] (see Checklist 1 for the full checklist).

### Setting

The study was undertaken in an Australian not-for-profit aged and community care organization. Research visits were conducted in participants’ homes. The study was conducted between March 2021 and April 2023.

### Participants

#### Phase 1: Consumer Advisory Group

Members of the organization’s existing Consumer Advisory Group (CAG) were invited to provide feedback on study procedures and the prototype device during a regular CAG meeting that was attended by the research team. The CAG comprised current or former home care recipients who met regularly to provide community perspectives on organizational and research activities. Participation was voluntary.

#### Phase 2: Community Participants

##### Recruitment

Participants were initially recruited through the aged and community care organization via study flyers distributed by home care nurses. Due to low recruitment, additional strategies included community presentations, flyers distributed through retirement living settings, social media notices, and word-of-mouth.

##### Inclusion and Exclusion Criteria

Eligible participants were aged 65 years or older, lived alone in metropolitan Melbourne, and used English as their primary spoken language. Living alone was required because the device aimed to capture external conversational contact rather than routine cohabiting conversation. People living alone are also more likely to experience problematic or enduring loneliness [28]. Participants were excluded if they had an acquired communication disorder (eg, aphasia, stroke, traumatic brain injury, or Parkinson disease; [29,30]), moderate-to-severe cognitive impairment, or severe mental illness that might compromise protocol adherence. Only English-speaking participants were eligible because the speech-detection algorithm was calibrated in English.

The original intention was to recruit 2 purposively sampled groups (total n=30), those experiencing loneliness (n=15) and those not experiencing loneliness (n=15), matched by age and gender, with quota sampling to include at least 30% (n=9) men. This strategy could not be achieved because of recruitment challenges that limited the final sample. Given the absence of comparable data, a formal sample size calculation was not feasible; the study therefore adopted a pragmatic feasibility approach based on team judgment and the number of available prototype devices.

### Materials: The CaT Pin Device

Prototype CaT Pin devices were developed by the RMIT Health Transformation Lab in conjunction with a product development company. The development comprised a 3-step process involving hard technology development, prototype development, and machine learning and algorithm training.

Twenty prototypes were produced, each with approximately 18 hours of battery life to support continuous wear throughout a typical day. The device was designed to capture time spent in conversation and store these data until they were transferred by the research team.

In this study, time spent in conversation was operationalized as device-detected speech from the wearer or nearby individuals. Periods without detected speech were also important, as detecting both conversational and nonconversational periods was necessary to explore whether conversation time could indicate loneliness. The prototype’s scale, shape, and wearing configuration are shown in Figure 1; device activity and charging indicators are shown in Figure 2. The device and clip were constructed from polyethylene terephthalate glycol and weighed approximately 93 g.

To protect the wearer’s privacy, the data collection algorithm was designed in such a way that the content of participants’ conversations was not captured and therefore could not be accessed by the researchers. The device collected all sounds that could be heard while it was being worn, with these sounds encrypted in real time such that listening to the encrypted sound files produced only white noise. All sound files were stored locally on the device and then downloaded by the research team at the end of the data collection period. Files could only be decrypted with the machine learning algorithm developed specifically for this project. Once the decryption key was entered into the algorithm, sounds were mapped onto 3 categories: speech from the wearer, speech from others, and all other noise. The algorithm then produced durations of speaking marked “own” for the wearer (participant) and “other” for other people speaking. These durations were then used to estimate the amount of conversation each participant engaged in while wearing the device.

**Figure 1.**
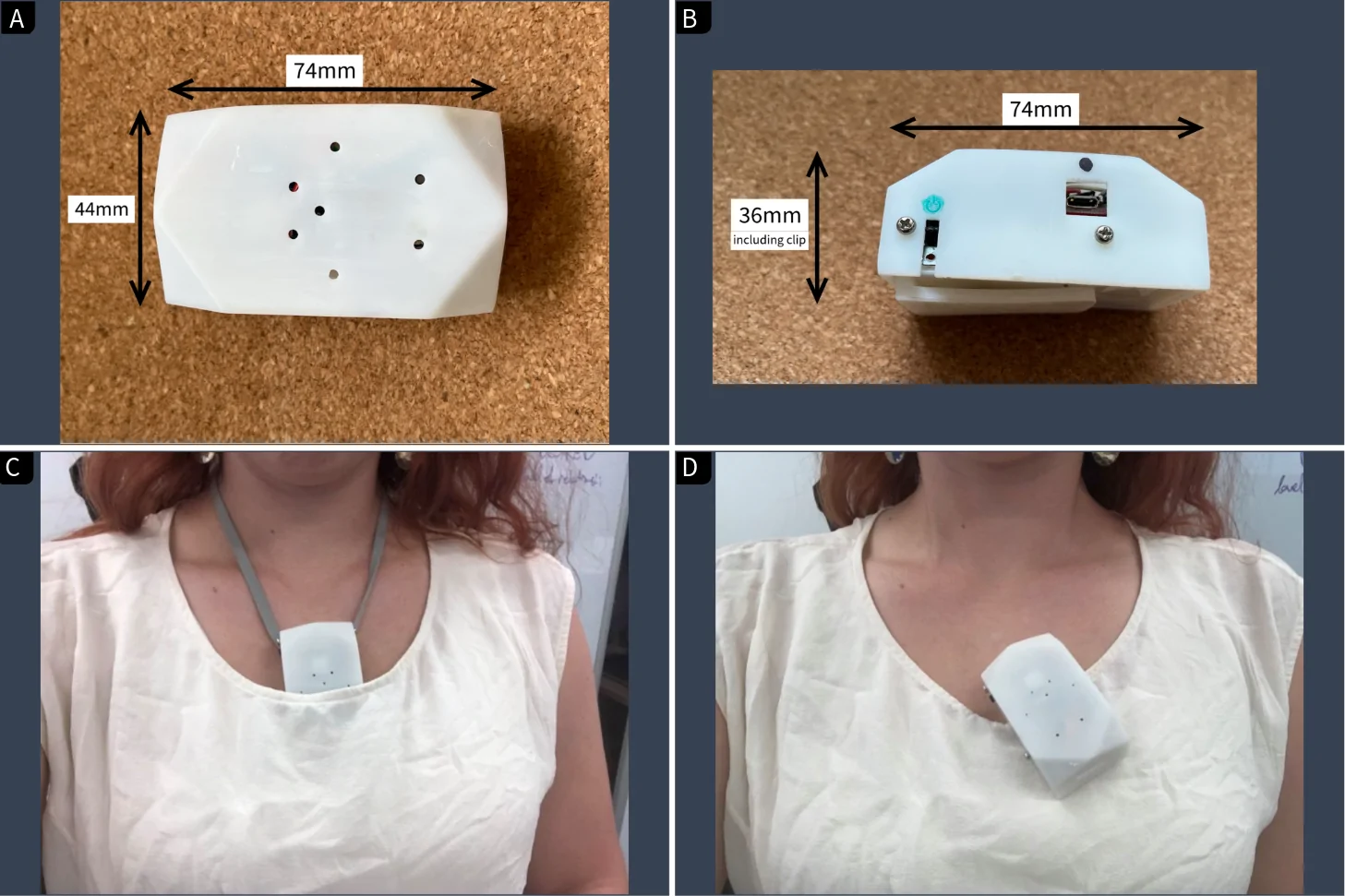
Working prototype of Conversation as Therapy (CaT) Pin. (A) and (B) show device measurements; (C) and (D) show the device being worn.

**Figure 2.**
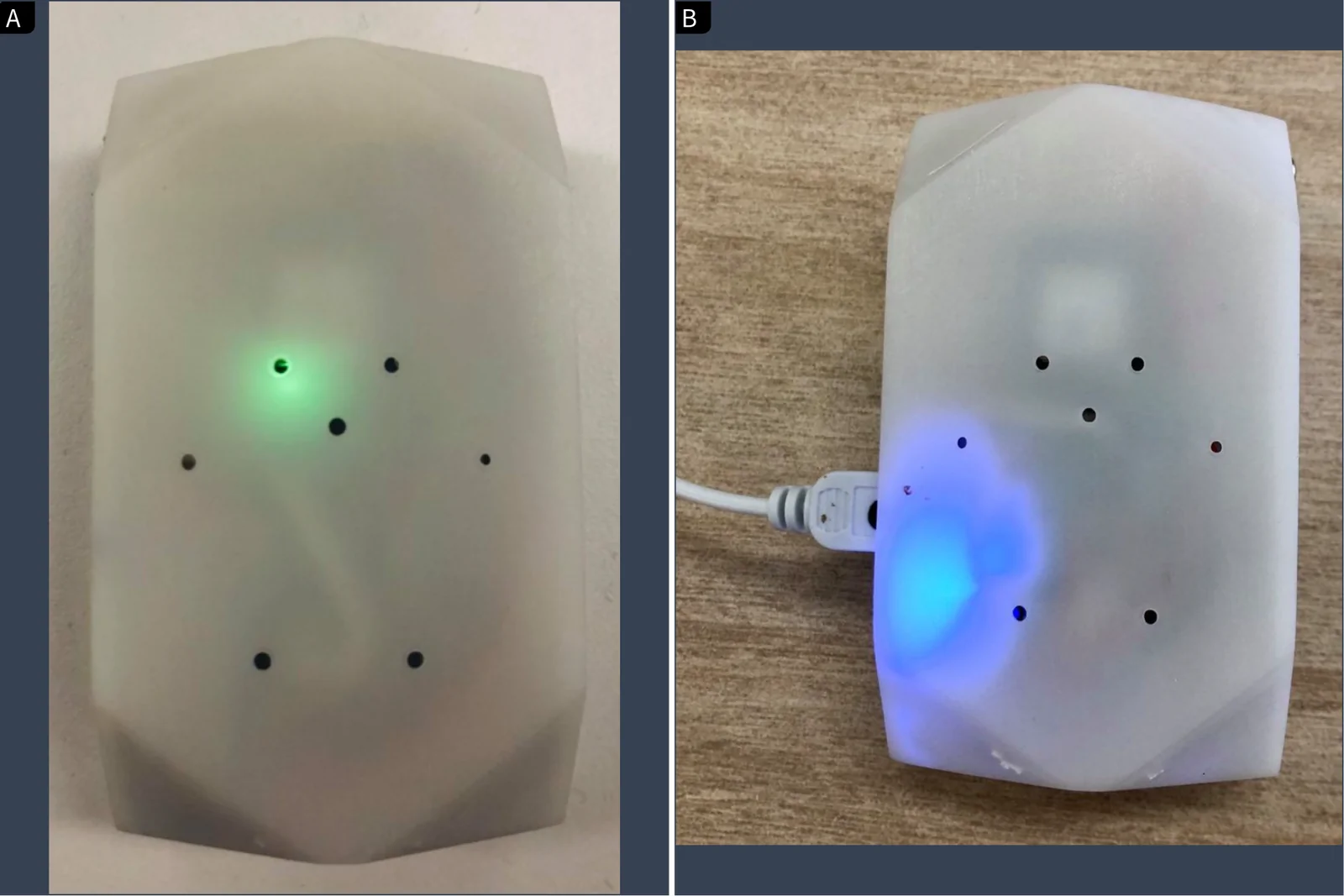
Prototype of Conversation as Therapy (CaT) Pin. (A) Turned on and recording, with a green light flashing every 5 seconds; (B) Plugged in to charge, with a blue light indicating it is charging.

### Procedures

#### Phase 1: Prototype Testing

In phase 1, the study procedures, activity log, and prototype device were presented to CAG members in a 1-hour discussion. Researchers documented recommended changes. Consenting participants then tested the device and activity log for 1 week, followed by a home-based semistructured interview with researchers (RO or GJ) about the process and experience. Interviews were audio-recorded and transcribed verbatim. A follow-up discussion was held with the CAG, where initial findings were shared and further feedback was invited. Feasible modifications to the study procedures, device, and activity log were implemented before phase 2.

#### Phase 2: Data Collection

##### Overview

Phase 2 used phase 1 learnings to assess whether device-derived conversation data and self-reported loneliness data could be collected and synchronized over 2 weeks. Figure 3 presents the participant flow. Data collection included a baseline visit, 2 weeks of device wear, activity log completion, and a follow-up semistructured interview when the device was collected. The research team contacted participants after commencement and at 1 week to answer questions and troubleshoot problems (see Multimedia Appendix 1 for an example).

**Figure 3.**
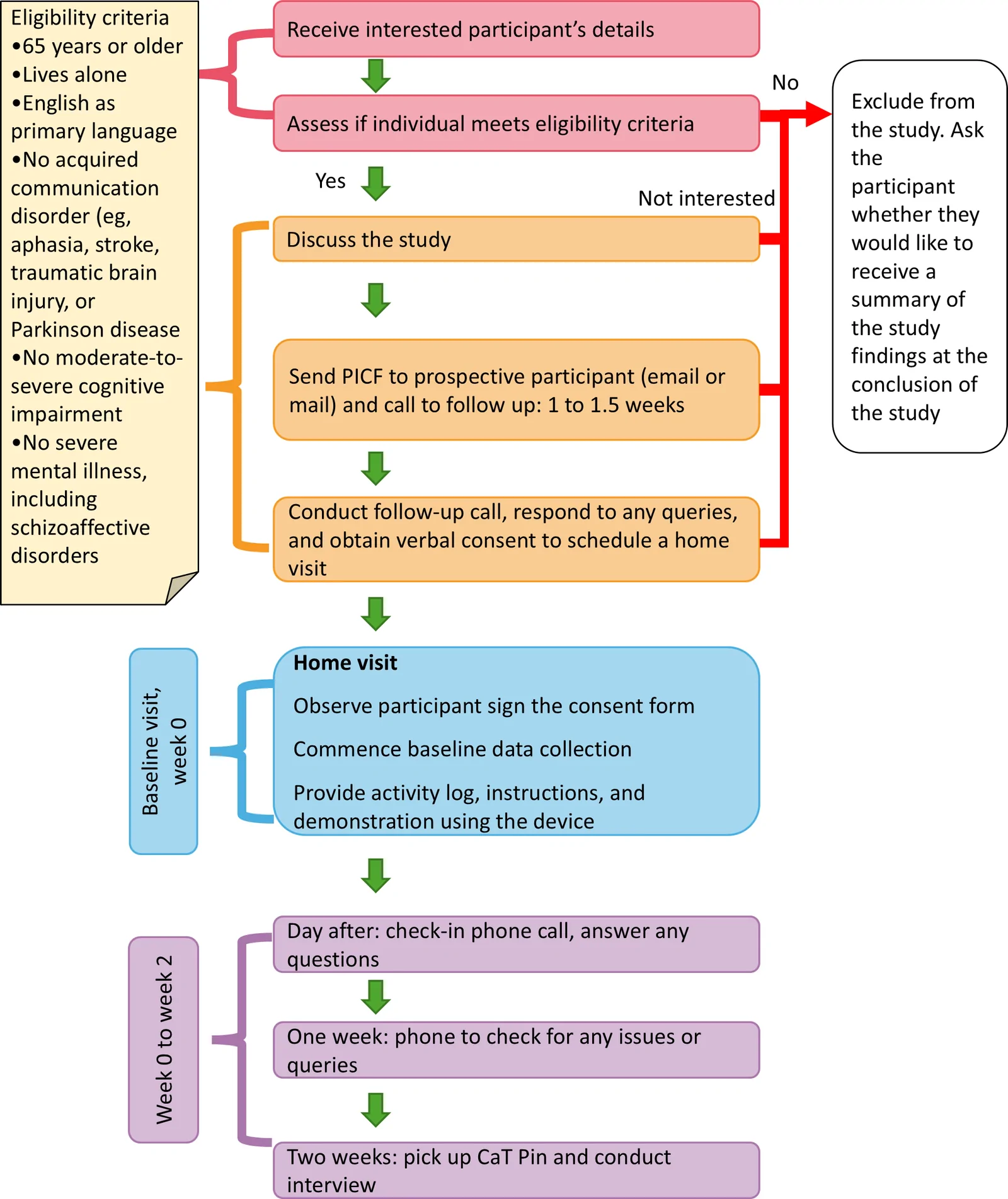
Proof-of-concept study participant flow. CaT Pin: Conversation as Therapy Pin; PICF: participant information and consent form.

##### Quantitative Measures

The planned primary outcome was self-reported loneliness, measured using an adapted direct single-item measure of loneliness [31] (How did you feel during this period? Likert scale: lonely none, a little, some, or all of the time). Participants recorded how they felt during each 6-hour interval over the 2-week study period. The planned exposure was time spent in conversation, derived from device-detected speech.

Because insufficient synchronized device-derived conversation data and self-reported loneliness data were generated, the planned exploration of the relationship between conversation time and loneliness could not be undertaken. Therefore, quantitative findings focus on descriptive feasibility outcomes, including device wear, activity log completion, data completeness, and the proportion of participants with synchronized data.

Participants also completed baseline questionnaires including demographic characteristics (age, gender, level of education, country of birth, and relationship status), measures of loneliness (UCLA 3-item Loneliness Scale [UCLA-3, 32]), social isolation (Lubben Social Network Scale-6 [LSNS-6, 33]), health-related quality of life (EQ-5D-5L [34] items), mental well-being (Short Warwick-Edinburgh Mental Well-Being Scale [SWEMWBS, 35]), and extraversion (Ten-Item Personality Inventory [TIPI, 36]), as well as information on health conditions, sensory impairment, informal care support, service use, and financial assistance. Detailed descriptions of measures and scoring procedures are provided in Multimedia Appendix 2.

##### Qualitative Measures

After 2 weeks, participants completed semistructured interviews (mean duration 38 min, range 21‐63 min) with the researcher (refer to Textbox 1 below for the interview guide).

Textbox 1. **Semistructured interview guide exploring loneliness, conversation, device usability, and acceptability.**

- What does being lonely mean to you? (results will be reported elsewhere)
- How did you feel wearing the CaT Pin device?
- How did you feel about the number of conversations you had? (with direct reference to activity log)
- What are your thoughts around using conversation levels to recognize loneliness?
- How and when would participants like a notification for intervention to occur if conversation levels dropped?

The interviews were conducted by a female postdoctoral qualitative researcher (RO) or an early-career researcher (GJ) who had conducted the baseline data collection and check-in calls but otherwise had no prior relationship with the participants. Interviews were audio-recorded and independently transcribed, with transcripts checked for accuracy against the recordings.

### Data Analysis

#### Phase 1

Due to its formative purpose of informing phase 2 and the small sample size, phase 1 qualitative data from interviews and notes were reviewed by RO and GJ using a pragmatic, structured, iterative process focused on identifying actionable items. Feedback and observations were identified and organized into key areas relating to the device, participant instructions, and activity log completion. Within each area, potential improvements, barriers, enablers, and user experiences were identified. Findings were discussed with the research team and subsequently refined through feedback from the CAG before being finalized.

#### Phase 2

##### Quantitative

Linear mixed models were originally planned to explore the association between time spent in conversation and loneliness.

Due to insufficient volumes of synchronized device and self-reported loneliness data, the planned inferential analyses were not undertaken. Analyses were therefore limited to descriptive statistics, including participant characteristics, device usage patterns, activity log completion, and data completeness. The proportion of participants with usable synchronized (paired) data was calculated to assess feasibility. All preparatory analyses were undertaken in R (version 4.3.1, R Foundation for Statistical Computing).

##### Qualitative

Reflexive thematic analysis was used to analyze qualitative data, involving an iterative and reflexive approach to the identification and interpretation of shared patterns of meaning across the dataset [37]. Analysis was facilitated by qualitative data management and analysis software NVivo (version 11, QSR International). Researchers adopted a constructionist epistemology with an experiential orientation, prioritizing the participants’ lived experience and interactions with the device while recognizing that meaning is actively coconstructed between participants and the researcher. Analysis was primarily inductive while remaining guided by the study aims and findings from phase 1.

Two researchers (RO and GJ) familiarized themselves with the data through repeated reading of transcripts, separately generating both semantic and latent codes. Researchers met face to face multiple times to discuss coding decisions, explore alternative interpretations, and collectively review and organize the codes according to shared meaning. Potential themes were developed through the organization of these code groups and were iteratively reviewed and refined collaboratively. Themes were then finalized, with representative quotes selected to illustrate each theme. Transcripts were not returned to participants for review; however, findings were summarized and shared with participants, and feedback was invited. The sample size was sufficient to develop a richly textured understanding of concepts from the participants’ data [38].

### Reflexivity

The research team had disciplinary backgrounds in health services research, aging, and digital health, with prior experience in qualitative research and analysis. Two researchers (RO and GJ) conducted data collection and analysis. Rapport was established with participants prior to the interviews through a baseline home visit and follow-up contacts during the 2-week study period, including device troubleshooting and procedural support. This provided the researchers with direct insight into participants’ ongoing experiences with the device, which could be explored further during interviews. Researchers adopted an empathetic and understanding approach during interviews, particularly when participants discussed device limitations, which facilitated open discussions of concerns about device use and its relevance to identifying loneliness.

Given this involvement, researchers actively considered how their own experiences with the device and its development, including negative perceptions of its usability and relevance formed while supporting participants, may have shaped data interpretation. Reflexivity was supported through ongoing open dialogue and discussion during analysis, in which assumptions and interpretations were critically examined in relation to the data. Care was taken to revisit transcripts to ensure that both confirming and contradictory patterns were attended to during theme development.

### Integration

Integration occurred during the interpretation and reporting of the findings, using a contiguous narrative approach. Quantitative feasibility outcomes (including data completeness, device wear, and data synchronization) were compared with qualitative findings (usability, acceptability, experiences, and conceptual validity). The research team compared findings across datasets to identify convergence and divergence, with qualitative data helping to explore and explain the quantitative feasibility challenges and data capture constraints.

### Ethical Considerations

Ethics approval for this study was provided by the Bolton Clarke Human Research Ethics Committee (approval number 210001) and the RMIT STEM College Human Ethics Advisory Network (approval number 24401). All participants received a verbal explanation of the study and written participant information, with time to ask questions before providing written informed consent. Participant privacy and confidentiality were maintained by assigning unique study identifiers, with data stored on secure, password-protected servers or in locked cabinets within the research team’s office. Participants did not receive compensation for their participation.

## Results

### Phase 1: Prototype Testing and Procedural Refinement

The initial CAG discussion (n=6, 3 women) and subsequent prototype testing (n=3, 1 women) generated recommendations across 3 broad areas: the device, participant instructions, and activity log completion. Device-related feedback focused primarily on wearability, comfort, security, visibility, charging, and ease of operation. Feedback regarding participant instructions highlighted the need for clearer explanations of device use, placement, troubleshooting, and study requirements. Activity log feedback focused on reducing burden, improving usability, clarifying loneliness response options, and capturing contextual information about conversations. Recommendations were reviewed with the CAG. Detailed recommendations are provided in Multimedia Appendix 3, including those that the research team was able to address. Feasible modifications were implemented before phase 2, although key design and functionality issues remained unresolved and subsequently affected feasibility.

### Phase 2: Recruitment and Sample Characteristics

#### Sample Characteristics

A total of 23 participants (15 women; mean age 79.4 y, SD 6.7 y) were recruited to phase 2, of whom 7 were classified as lonely. Recruitment through the aged care organization yielded 6 participants over 6 months. Reported barriers identified by nurses included ineligibility due to cognitive impairment or mental health conditions, limited English proficiency, or lack of interest in participation. Additional community recruitment improved enrollment, but the planned matched sampling by loneliness, age, and gender was not achieved. These challenges highlight feasibility constraints in recruiting older adults for wearable technology-based research, particularly where eligibility criteria, perceived burden, and contextual factors, such as COVID-19, intersect.

Participant characteristics are presented in Table 1. The final sample comprised individuals with relatively high education levels, living in predominantly socioeconomically advantaged areas, yet a substantial proportion relied on government support. Participants also reported a range of functional limitations, including problems with mobility and usual activities, alongside common sensory challenges such as vision and hearing impairments.

Because insufficient synchronized data were generated, planned inferential analyses were not undertaken. Descriptive findings are, therefore, reported as feasibility outcomes and indicate high variability in device wear, low completion of repeated loneliness measures, and insufficient synchronized data to support the exploration of the relationship between conversation time and loneliness.

**Table 1.** Participant characteristics for phase 2 of feasibility trial (N=23).

Characteristics	Value
Age (y), mean (SD)	79.4 (6.7)
Gender, women, n (%)	15 (65.2)
Highest education level completed, n (%)	
Primary school	4 (17.4)
High school	6 (26.1)
Vocational training	5 (21.7)
Undergraduate degree	3 (13.0)
Postgraduate degree (including doctorate)	5 (21.7)
Country of birth, Australia, n (%)	14 (60.9)
Relationship status, n (%)	
Widowed	16 (69.6)
Divorced	4 (17.4)
Never married	3 (13.0)
Socially isolated^a^, n (%)	6 (26.1)
Had an informal caregiver or caregivers, n (%)	7 (30.4)
In receipt of support, n (%)^b^	
Government pension (aged/Department of Veterans’ Affairs)	17 (73.9)
Aged care service	12 (52.2)
Index of Relative Socioeconomic Advantage and Disadvantage (IRSAD) quintile, n (%)	
1 (decile 1‐2; most disadvantage)	0 (0)
2 (decile 3‐4)	0 (0)
3 (decile 5‐6)	3 (13.0)
4 (decile 7‐8)	4 (17.4)
5 (decile 9‐10; least disadvantage/most advantage)	16 (69.6)
Level of mental well-being^c^, n (%)	
High	5 (21.7)
Moderate	16 (69.6)
Low	2 (8.7)
Mental health-related diagnosis, yes, n (%)	7 (30.4)
Any problems with...^d^, n (%)	
Mobility	13 (56.5)
Self-care	5 (21.7)
Usual activities	10 (43.5)
Pain/discomfort	19 (82.6)
Anxiety/depression	12 (52.2)
Hearing problems, n (%)	8 (34.8)
Vision problems, n (%)	18 (78.3)
Self-assessed general health^e^, median (IQR)	85 (50-90)
Extraversion^f^, n (%)	14 (60.9)

a Socially Isolated, measured as scoring <12 on the Lubben Social Network Scale-6 [33].

b Participants could indicate all applicable options; therefore, percentages may exceed 100%.

c Levels of mental well-being from the Short Warwick-Edinburgh Mental Well-Being Scale [35].

d Measured using EQ-5D-5L (health-related quality of life) [34].

e EuroQol Visual Analogue Scale (EQ-VAS, on a scale of 0‐100) [34].

f Extraversion was measured using 2 items from the Ten-Item Personality Inventory (TIPI) [36].

#### Feasibility Outcomes: Device Wear, Activity Log Completion, and Data Synchronization

A primary feasibility question was whether participants generated sufficient synchronized device-derived conversation data and self-reported loneliness data to support exploratory analysis. Table 2 below provides a summary of feasibility outcomes.

**Table 2.** Feasibility outcomes: device use, activity log completion, and data synchronization phase 2, N=23).

Feasibility domain	Indicator	Result, n/N, (%)	Key issue identified
Recruitment	Participants enrolled	23/30 (not achieved)	Recruitment lower than planned; difficulty engaging eligible participants
Device uptake	Wore device at least partially	21/23 (91.3)	Two participants stopped wearing entirely due to burden, comfort, and usability issues
Selective device use	Removed device during specific activities	14/23 (60.9)	Removed during sleep, meals, activities in which wearing device would be uncomfortable or unsafe, or when not expecting conversation
Activity log completion	Adequate completion across time periods	Not achieved	Missing data across all time blocks, particularly evenings/overnight
Data synchronization	Generated sufficient synchronized device + log data	8/23 (34.8)	Insufficient for planned analysis
Primary feasibility outcome	Achieved sufficient data to test research questions	Not achieved	Low synchronization due to combined device, participant, and data architecture issues
Sources of missing data	Primary contributors to incomplete data	Not applicable	Inconsistent wear, incomplete logs, temporal misalignment, and misunderstanding of study requirements

##### Device Use

Twenty-one of 23 participants wore the device at least partially over the 2-week period. However, device wear was inconsistent: 2 participants stopped wearing the device entirely, and almost two-thirds removed it during daily activities, for example, during vigorous activity or exercise, meals, clothes shopping, sleep, medical appointments or care activities, or when alone and not conversing with others.

##### Activity Log Completion

Completion of activity logs was variable, with missing entries across all time periods and particularly high levels of missingness during the evening and overnight periods.

##### Synchronized Data

Only 8 out of 23 participants generated sufficient synchronized device-derived conversation data and self-reported loneliness data for potential analysis, which was insufficient to explore the relationship between time spent in conversation and loneliness.

Missing data arose from multiple interacting sources: inconsistent device wear, incomplete activity log entries, misalignment between continuous device data and categorized time blocks, and misunderstanding of study requirements. Many participants interpreted the device as capturing active conversation only, rather than both conversation and silence. As continuous wear became burdensome, device wear was often prioritized during anticipated speaking periods. The research team also did not anticipate the importance of reinforcing nonconversational wear or the extent to which burden would affect adherence.

### Qualitative Findings

Due to insufficient synchronized data, the qualitative findings became particularly important in understanding the feasibility challenges, device acceptability, and conceptual issues identified. Three main themes were identified from participant interviews (n=23): conceptual mismatch, design barriers, and functional limitations.

#### Conceptual Mismatch: Limitations of Conversation Quantity as an Indicator of Loneliness

Participants broadly understood and supported the concept of addressing loneliness but questioned whether the quantity of conversations is a meaningful indicator of loneliness. For some, “the number and amount of conversations I have really isn’t something that makes a difference to me” (P2_10). Others stated that they preferred to listen rather than talk. Furthermore, it was not the quantity of conversations, but rather the quality that was relevant, as described in the following quote:

*It all depends who that person is, because if it is one who has stimulated you immensely, you’ll care, and wear it all day long, and still think about it. If it’s someone who said “Lovely weather today love” that doesn’t make much impact, but it really is the person.*

[P2_12]

When considering triggering contact with a nominated person, again, it was important to participants that this be tailored to the individual. Most thought that face-to-face contact would be most ideal, but others shared that a phone call or a text message would also be feasible or even preferred.

*Or a text message, if they are tech savvy. Because that’s not as intrusive as a phone call. I think face to face is probably better. But phone calls are important too.*

[P2_11]

The relationship with the nominated person was seen as critical, with participants articulating that it needed to be the right person, someone they knew, could engage with, and had a real connection with. The majority stated that it should not be an unknown volunteer, but rather a personal friend, stemming from a desire for mutual reciprocity rather than obligation.

*I know I would know in the back of my mind well, that’s their job… unless it was a friend.*

[P2_18]

Interestingly, despite the acknowledged need for a way to identify when someone was experiencing loneliness to enable timely interventions, many participants intimated similar sentiments, “That might work for some, not all, But I don’t think I have the need for it,” (P2_4) often because they felt they already had the level of interaction that they needed. Participants did identify that the device might be a potential safety net for people who do not get as much social contact, are estranged from family members, or are lonely. Some of these participants stated that they would engage as the nominated person on the other end of an alert, supporting someone who wants conversation.

These findings highlight a fundamental conceptual limitation, in that conversation quantity may not function as a standalone proxy for loneliness and may require integration with more nuanced, relational measures.

#### Design Barriers: A Device That Does Not Fit the Body or Daily Life Drives Selective Use

The physical design of the device was not deemed acceptable and was a major barrier to sustained use for all 23 participants. Overall, the prototype was considered too large, heavy, and clunky, with sharp edges; as P2_1 noted, “It’s like wearing a brick around your neck,” similarly described as “annoying,” “a cumbersome thing,” “awkward to wear,” and something that “got in the way.”

Some participants stopped wearing the device entirely (n=2), while others selectively removed it during activities (n=14). The design meant that participants chose not to wear the device when they knew they were not going to engage in any conversations, as it was “a pain in the you-know-what to have to wear it” (P2_15). Participants also reported concerns related to visibility (eg, flashing light), aesthetics and stigma, and interference with daily activities, as described by the participant below.

*When I was going out, and I went past the mirror and I could see it blinking! [LAUGHTER] I was going somewhere special so I turned it back [to front]. I’m not going out with that light flashing on and off and everything!*

[P2_08]

The use of a lanyard to compensate for clip failure further reduced comfort and usability.

Some participants were particularly concerned about the lack of consideration given to design and strongly recommended that, in the future, biomedical engineers consider design before testing any device with participants.

*…there is no reason why [the device developer couldn’t] do something that was body accessible, body aware... the machine is just as capable of making something which contours to the body and fits the body rather than something which is square and hard angles, and always uncomfortable. I was never not aware that I had it on. If they had designed the outer casing, it doesn’t matter how big it is, but the outer casing, so that it had smooth curves, and it was compatible with the body, and it had some sort of human interaction in it rather than just. Well, “this is what we're going to do. So make a nice little square box to put it in”. It shows no thinking, no understanding of design, and as a designer that really bugged me*.

[P2_07]

For those with other medical assistance devices (eg, medical alert, smart devices, or falls pendants), comparisons were made with the less obtrusive design of those other devices and their better integration into participants’ lifestyle.

*If it was something smaller. And you aren’t conscious of it swinging around your neck, so you don’t notice it. Like this thing here. I have a wrist band, like a watch, and this is fine.*

[P2_04]

The participants were unanimous that device design significantly affected engagement, leading to reduced wear time and directly impacting data completeness. Usability and aesthetics emerged as critical determinants of feasibility.

#### Functional Limitations: Poor Usability and Reliability Reduce Trust and Sustained Use

Participants reported multiple acceptability issues related to device functionality, that is, how well the device worked and how users could interact with it. Although the device was made specifically for use by older community members, some components were not practical for older adults with reduced dexterity or people with less acute vision.

The main issues were the small and fiddly charging port and the on/off switch. Both were crucial for the device to be used successfully; however, many participants had difficulty with the prototype configuration.

*The thing that fits into this here [the charger], doesn’t go in properly*

[P2_5]

*If someone had no nails, I think they’d have trouble [turning it on and off].*

[P2_1]

There were numerous malfunctions with the device, particularly with the light that indicated whether it was working or charging. As a result, some participants wore the device but were concerned or unaware that it was not collecting data correctly, were disconcerted when the lights went off following charging, or had to switch devices partway through the trial. Device failures required replacements during the study in some cases, further disrupting data collection.

*It was ok for the first 10 days or so, at the start, and in the last few days it started to play up. I think the battery is not good.*

[P2_11]

*Half the day it started and then at about 4:00 o'clock, it started flashing red.*

[P2_14]

Despite the troubleshooting and extensive support from the research team, participants were also concerned about using the device “correctly.”

*Well, sometimes I used to wonder if it was actually a recording, because I don’t know if I had it in the right place, and I thought it was here, and I thought maybe that doesn’t even record*

[P2_2]

Functional limitations reduced trust in the device and increased participant burden, contributing to inconsistent use and incomplete data.

### Integration

Integration of quantitative and qualitative findings demonstrated that low levels of synchronized data were not solely attributable to technical issues. Quantitative findings identified low levels of synchronized data and inconsistent device wear, while qualitative findings helped explain these outcomes through participant burden, selective device use, usability concerns, and misunderstandings regarding study requirements. Across both datasets, 3 interrelated categories of feasibility constraints were identified. First, device-related constraints, including design, comfort, and functionality, limited sustained device wear. Second, participant-related constraints affected data capture, including misunderstanding of study requirements and selective device use. Third, data architecture constraints, including misalignment between continuous device-derived data and categorical activity log entries, contributed to incomplete datasets. Together, these factors resulted in insufficient synchronized data to enable exploration of the relationship between time spent in conversation and loneliness.

## Discussion

### Feasibility Findings

This mixed methods feasibility study evaluated whether the CaT Pin could capture time spent in conversation among older adults living alone and whether sufficient synchronized device-derived conversation data and self-reported loneliness data could be generated to enable exploration of the relationship between time spent in conversation and loneliness. Following phase 1 refinement of the device and procedures, phase 2 evaluated feasibility and acceptability among community-dwelling older adults. In its current form, this approach was not feasible. The integrated findings identified interrelated device, participant, and data architecture barriers that prevented the generation of sufficient synchronized data.

#### Device Usability and Real-World Adoption

As technologies designed to promote well-being in older adults become more common, wearable devices are expected to be functional, appealing, and integrated into everyday life [25]. Device design affected recruitment and engagement in this study, consistent with previous research [26]. Participants consistently described the prototype as too large, heavy, and visually conspicuous, and many limited their use because it was uncomfortable, intrusive, or incompatible with daily activities. These issues directly affected feasibility by reducing wear time, increasing selective nonuse, and ultimately limiting analyzable data.

The findings reinforce prior work, indicating that devices for older adults must be comfortable, discreet, and easy to use to support sustained engagement [25,26]. As Moore et al [26] observed, “Any device should serve its purpose, and the primary function of the device should be reliable and easy to use; cause minimal disruption in users’ lives, be aesthetically pleasing, and should not draw attention to the user or single them out as a patient.” The present findings suggest that these expectations apply even during feasibility testing. A technically promising prototype may fail before the underlying concept can be evaluated if it is uncomfortable, unattractive, or difficult to operate.

These findings also have implications for co-design. Although engineers, clinical researchers, and older adults contributed to the later stages of device and protocol development, older adults were not involved in initial ideation and design [2]. Persistent usability concerns suggest that participation at later stages was insufficient. Human-centered design approaches emphasize involving end users early and iteratively so assumptions about aesthetics, wearability, and usability can be challenged before major development decisions are made [2,26,39,40]. In this study, consumer feedback improved aspects of the protocol and device, but resource limitations prevented substantive redesign. Earlier and more meaningful involvement of older adults as design partners in both ideation and design [2] may increase both acceptability and the likelihood of achieving study aims while reducing unintended ageism in technology development [5,26].

#### Data Capture Feasibility and Synchronization

A second major finding relates to the measurement architecture required to test the study concept. The study was not undermined by device malfunction or an isolated adherence issue but by the interaction between continuous device-generated data, categorical activity-log data, and participant interpretation of what needed to be recorded.

The device produced continuous, time-stamped speech data, whereas the activity log required participants to summarize activities and loneliness in 6-hour blocks. Although device data were later grouped into these periods, temporal precision was reduced and interpretation was constrained. High rates of missing data, particularly in evening and overnight periods, further limited synchronization. Researchers were also unable to access device data in real time, meaning that device problems and insufficient data capture were not identified until the study had concluded.

Participant behavior added further complexity. Many participants interpreted the device as measuring conversation only and therefore did not wear it during periods when they did not expect to speak. However, both conversation and silence were important to evaluating the study concept. Researchers were also cautious in enforcing high levels of device wear because of concern that participants would cease wearing it altogether. These findings highlight a key challenge in passive sensing research: participants engage with devices according to their own understanding of what is important. Future studies will require clearer communication about the importance of capturing both event and nonevent time, alongside systems that enable real-time monitoring and correction of data capture problems. More broadly, feasibility depends not only on technical capability but also on alignment between the technology, protocol, and participant understanding.

#### Conceptual Implications for Loneliness Detection

The qualitative findings raise important conceptual implications. While the device was designed to quantify time spent in conversation, participants frequently questioned whether conversational quantity was meaningful in itself. Instead, they emphasized the quality, meaning, and personal relevance of interactions. As one participant observed, a brief interaction with the “right” person could be far more significant than an extended casual conversation. This challenges the premise that conversation time alone can function as a sufficient indicator of loneliness.

Identifying appropriate technological markers of loneliness remains challenging [41]. Loneliness is subjective [42] and multidimensional and is influenced by factors at the individual, interpersonal, community, and societal levels [43]. Quantitative findings also suggest that measurement strategy and participant burden strongly influence feasibility. The single-item loneliness measure may have lacked sensitivity to capture the dynamic nature of loneliness in this context and may have been influenced by participant interpretation or reporting bias related to stigma [44]. Our findings suggest that conversation quantity may be one component within a broader multidimensional model rather than a standalone indicator. This aligns with literature emphasizing the cognitive, relational, and situational complexity of loneliness [42,43,45].

The findings also highlight the potential role of stigma. Participants often viewed the device as something that might benefit “other people” rather than themselves. In parallel, the visible and somewhat clinical appearance of the prototype may have amplified concerns associated with wearing a health-related device in public [46,47]. Future technology-enabled approaches to loneliness will therefore need to consider not only measurement validity but also identity, dignity, and the social meanings attached to both loneliness and wearable devices.

### Implications for Future Research

First, prototypes must be functional, acceptable, and integrated into everyday life, with attention to comfort, dexterity, vision, and aesthetics [25,26]. Earlier involvement of older adults in both ideation and design is likely to improve alignment between technology and user needs [2,26,40].

Second, generating synchronized data requires careful planning and alignment between technological and self-report measures. Future studies should incorporate real-time monitoring to detect device malfunctions, missing data, and misunderstandings of study procedures before data collection is complete.

Third, loneliness is unlikely to be reducible to a single passive behavioral metric. Future research should adopt more conceptually nuanced approaches that recognize loneliness as subjective and multidimensional [41,42]. Conversation time may be better understood as one component within a broader framework that includes self-report, context, personal meaning, and individual preferences [41-43,45].

Finally, this study demonstrates the value of transparently reporting negative feasibility findings. Although extensive refinement occurred within the available resources, device-related barriers were directly reflected in the quantity and quality of data collected. Reporting these challenges provides practical guidance for researchers, developers, and funders designing future digital health technologies for older adults.

### Strengths and Limitations

#### Methodological Strengths

A key strength of this study was demonstrating that the concept for a device such as the CaT Pin cannot be separated from its usability and acceptability. To address this novel research question, we adopted a pragmatic mixed methods approach, triangulating quantitative and qualitative findings to provide a comprehensive assessment of feasibility, including device performance, participant experience, and data capture processes. A further strength was the inclusion of older adults in prototype testing and refinement, ensuring end-user perspectives informed evaluation of both the device and study procedures.

#### Limitations

This study has several limitations, grouped into device-related, procedural, and conceptual domains. Recruitment was a significant challenge. Although 30 participants were planned (15 lonely and 15 not lonely), only 23 were recruited (of whom 7 were lonely). Consequently, the intended matched cohort design could not be achieved. The final sample was relatively small, unmatched, and predominantly from socioeconomically advantaged areas, limiting the broader generalizability of the findings. These feasibility barriers may be even more pronounced in socioeconomically disadvantaged or less technologically engaged populations. However, the primary purpose of the study was to identify feasibility barriers affecting implementation and measurement rather than to estimate effects or test associations.

#### Device-Related Limitations

Limited project funding constrained device development and refinement. The prototype’s size, weight, and functional instability reduced sustained wear and directly contributed to incomplete data capture despite iterative refinement. These issues, particularly the device’s weight and bulk, influenced both the willingness to wear the device and the consistency of use, contributing to the limited amount of usable quantitative data captured.

#### Procedural and Implementation Limitations

Procedural limitations included recruitment difficulties, variable adherence to the study procedures, and reliance on self-reported activity logs, which were affected by missing data and participant interpretation. The challenges in synchronizing continuous device-derived data with participant-reported activity logs also highlight limitations inherent in combining passive sensing technologies with self-reported measures. Despite assurances that conversational content was not accessible, some potential participants expressed concerns regarding privacy and possible “eavesdropping,” consistent with previous research on wearable technologies [46,47]. Concerns about data breaches and scams may also have reduced willingness to participate.

The project commenced while the impacts of the COVID-19 pandemic were still being experienced, and concerns about virus exposure and social reengagement may have further affected recruitment. In addition, the lack of real-time access to device data meant that missingness, malfunction, and misunderstanding of study requirements could not be identified and addressed during the trial and were only fully recognized after data collection had ended.

#### Conceptual Limitations

A conceptual limitation was the assumption that time spent in conversation could function as an indicator of loneliness. Participant accounts suggested that the quality and meaning of interaction were more relevant to loneliness than conversational quantity.

Taken together, these limitations suggest that the inability to explore the original concept reflects not only practical feasibility barriers but also possible limitations in the underlying conceptual model. This is an important finding, indicating that future technological approaches to loneliness detection may need to move beyond single passive behavioral indicators and engage more directly with the subjective and multidimensional nature of loneliness.

### Conclusions

This mixed methods feasibility study showed that sufficient synchronized device-derived conversation data and self-reported loneliness data were not generated to enable exploration of the relationship between time spent in conversation and loneliness among older adults living alone. However, the principal contribution of the study lies not in testing the relationship but in demonstrating that the concept for a device such as the CaT Pin cannot be divorced from its usability and acceptability; to generate useful data, wearable technologies must be not only functional but also comfortable, discreet, and acceptable to intended users. Conceptually, the study suggests that time spent in conversation is unlikely to function as a standalone indicator of loneliness. Participants consistently emphasized the quality, meaning, and personal relevance of social interactions, highlighting the need for more nuanced and multidimensional approaches to loneliness detection. Future research should explore measurement models that combine behavioral indicators with contextual and subjective experiences of loneliness and should incorporate real-time monitoring and better alignment between passive sensing and self-report measures.

Taken together, these findings provide practical guidance for the design and evaluation of digital health and wearable technologies intended for older adults while highlighting the importance of aligning technological innovation with the lived experience of intended users.

## Supplementary material

10.2196/94027 Multimedia Appendix 1 Activity and conversation log.

10.2196/94027 Multimedia Appendix 2 Baseline measures and scoring procedures.

10.2196/94027 Multimedia Appendix 3 Full recommendations for improvements on the device, instructions for user guide, and the activity log from phase 1 feedback and testing with older community members.

10.2196/94027 Checklist 1 Completed GRAMMS checklist.

## Abbreviations

CAG: Consumer Advisory Group
CaT: Conversation as Therapy
GRAMMS: Good Reporting of a Mixed Methods Study
LSNS-6: Lubben Social Network Scale-6
SWEMWBS: Short Warwick-Edinburgh Mental Well-being Scale
TIPI: Ten-Item Personality Inventory

## References

[1] Zhang M (2023). Older people’s attitudes towards emerging technologies: a systematic literature review. Public Underst Sci.

[2] Sumner J, Chong LS, Bundele A, Wei Lim Y (2021). Co-designing technology for aging in place: a systematic review. Gerontologist.

[3] Lee C (2022). Technology and aging: the jigsaw puzzle of design, development and distribution. Nat Aging.

[4] Chang WC, Lin WZ, Wang HD (2023). Aesthetics model applied for older adults: daily life assistive device study. Phys Occup Ther Geriatr.

[5] Mannheim I, Wouters EJM, Köttl H, van Boekel LC, Brankaert R, van Zaalen Y (2023). Ageism in the discourse and practice of designing digital technology for older persons: a scoping review. Gerontologist.

[6] (2024). Empowering older Australians with better product usability. https://www.gcma.net.au/better-product-usability-report.

[7] Badcock JC, Holt-Lunstad J, Garcia E, Bombaci P, Lim MH (2022). Position statement: addressing social isolation and loneliness and the power of human connection. https://www.gilc.global/_files/ugd/410bdf_74fffc2d18984b0e8217288b1b12d199.pdf.

[8] Lim MH, Rodebaugh TL, Zyphur MJ, Gleeson JFM (2016). Loneliness over time: the crucial role of social anxiety. J Abnorm Psychol.

[9] Hämmig O (2019). Health risks associated with social isolation in general and in young, middle and old age. PLoS One.

[10] Holt-Lunstad J (2021). Loneliness and social isolation as risk factors: the power of social connection in prevention. Am J Lifestyle Med.

[11] Wang S, Molassiotis A, Guo C, Leung ISH, Leung AYM (2023). Association between social integration and risk of dementia: a systematic review and meta-analysis of longitudinal studies. J Am Geriatr Soc.

[12] Lazzari C, Rabottini M (2022). COVID-19, loneliness, social isolation and risk of dementia in older people: a systematic review and meta-analysis of the relevant literature. Int J Psychiatry Clin Pract.

[13] Hakulinen C, Pulkki-Råback L, Virtanen M, Jokela M, Kivimäki M, Elovainio M (2018). Social isolation and loneliness as risk factors for myocardial infarction, stroke and mortality: UK Biobank cohort study of 479 054 men and women. Heart.

[14] Long RM, Terracciano A, Sutin AR (2023). Loneliness, social isolation, and living alone associations with mortality risk in individuals living with cardiovascular disease: a systematic review, meta-analysis, and meta-regression. Psychosom Med.

[15] Lim M, Lambert G, Thurston L, Argent T, Eres R, Qualter P (2020). Survey of health and wellbeing – monitoring the impact of COVID-19. https://www.swinburne.edu.au/media/swinburneeduau/research-institutes/iverson-health/Loneliness-in-COVID-19-15-07-20_final.pdf.

[16] Prohaska T, Burholt V, Burns A (2020). Consensus statement: loneliness in older adults, the 21st century social determinant of health?. BMJ Open.

[17] Barreto M, van Breen J, Victor C (2022). Exploring the nature and variation of the stigma associated with loneliness. J Soc Pers Relat.

[18] Wu C, Barczyk AN, Craddock RC (2021). Improving prediction of real-time loneliness and companionship type using geosocial features of personal smartphone data. Smart Health.

[19] Jafarlou S, Azimi I, Lai J (2024). Objective monitoring of loneliness levels using smart devices: a multi-device approach for mental health applications. PLoS One.

[20] (2015). Evidence review: loneliness in later life. https://www.ageuk.org.uk/globalassets/age-uk/documents/reports-and-publications/reports-and-briefings/health--wellbeing/rb_june15_lonelines_in_later_life_evidence_review.pdf.

[21] Mehl MR, Vazire S, Holleran SE, Clark CS (2010). Eavesdropping on happiness: well-being is related to having less small talk and more substantive conversations. Psychol Sci.

[22] Milek A, Butler EA, Tackman AM (2018). “Eavesdropping on happiness” revisited: a pooled, multisample replication of the association between life satisfaction and observed daily conversation quantity and quality. Psychol Sci.

[23] Sun J, Harris K, Vazire S (2020). Is well-being associated with the quantity and quality of social interactions?. J Pers Soc Psychol.

[24] Sandstrom GM, Dunn EW (2014). Social interactions and well-being: the surprising power of weak ties. Pers Soc Psychol Bull.

[25] Lewis JE, Neider MB (2017). Designing wearable technology for an aging population. Ergonom Des.

[26] Moore K, O’Shea E, Kenny L (2021). Older adults’ experiences with using wearable devices: qualitative systematic review and meta-synthesis. JMIR mHealth uHealth.

[27] O’Cathain A, Murphy E, Nicholl J (2008). The quality of mixed methods studies in health services research. J Health Serv Res Policy.

[28] Barjaková M, Garnero A, d’Hombres B (2023). Risk factors for loneliness: a literature review. Soc Sci Med.

[29] Cummings L (2023). Communication disorders: a complex population in healthcare. Lang Health.

[30] Aldridge D, Cahill L, Theodoros D (2023). Assessment of communication competence in acquired communication disorders: a systematic scoping review. Int J Speech Lang Pathol.

[31] (2018). Measuring loneliness: guidance for use of the national indicators on surveys. https://www.ons.gov.uk/peoplepopulationandcommunity/wellbeing/methodologies/measuringlonelinessguidanceforuseofthenationalindicatorsonsurveys.

[32] Hughes ME, Waite LJ, Hawkley LC, Cacioppo JT (2004). A short scale for measuring loneliness in large surveys: results from two population-based studies. Res Aging.

[33] Lubben J, Blozik E, Gillmann G (2006). Performance of an abbreviated version of the Lubben Social Network Scale among three European community-dwelling older adult populations. Gerontologist.

[34] Herdman M, Gudex C, Lloyd A (2011). Development and preliminary testing of the new five-level version of EQ-5D (EQ-5D-5L). Qual Life Res.

[35] Stewart-Brown S, Tennant A, Tennant R, Platt S, Parkinson J, Weich S (2009). Internal construct validity of the Warwick-Edinburgh Mental Well-being Scale (WEMWBS): a Rasch analysis using data from the Scottish Health Education Population Survey. Health Qual Life Outcomes.

[36] Gosling SD, Rentfrow PJ, Swann WB (2003). A very brief measure of the Big-Five personality domains. J Res Pers.

[37] Braun V, Clarke V (2021). Thematic Analysis: A Practical Guide.

[38] Braun V, Clarke V (2016). (Mis)conceptualising themes, thematic analysis, and other problems with Fugard and Potts’ (2015) sample-size tool for thematic analysis. Int J Soc Res Methodol.

[39] Melles M, Albayrak A, Goossens R (2021). Innovating health care: key characteristics of human-centered design. Int J Qual Health Care.

[40] Mannheim I, Schwartz E, Xi W (2019). Inclusion of older adults in the research and design of digital technology. Int J Environ Res Public Health.

[41] Site A, Lohan ES, Jolanki O (2022). Managing perceived loneliness and social-isolation levels for older adults: a survey with focus on wearables-based solutions. Sensors (Basel).

[42] Skoko A, Seewer N, Mund M, Krieger T (2025). Revisiting the cognitive and behavioral aspects of loneliness: insights from different measurement approaches. PLoS One.

[43] Ogrin R, Cyarto EV, Harrington KD (2021). Loneliness in older age: what is it, why is it happening and what should we do about it in Australia?. Australas J Ageing.

[44] Neves BB, Petersen A (2025). The social stigma of loneliness: a sociological approach to understanding the experiences of older people. Sociol Rev.

[45] Mansfield L, Victor C, Meads C (2021). A conceptual review of loneliness in adults: qualitative evidence synthesis. Int J Environ Res Public Health.

[46] Garcia Reyes EP, Kelly R, Buchanan G, Waycott J (2023). Understanding older adults’ experiences with technologies for health self-management: interview study. JMIR Aging.

[47] Canali S, Ferretti A, Schiaffonati V, Blasimme A (2024). Wearable technologies for healthy ageing: prospects, challenges, and ethical considerations. J Frailty Aging.

