# Supplementary material for: Feasibility of Using a Wearable Device to Identify Loneliness Among Older Adults Living Alone: Mixed Methods Study
Source: JMIR Form Res. 2026 Oct 15;10:e94027. doi: 10.2196/94027 (PMC13671184; doi:10.2196/94027)
Supplement: Supplementary file 2 [file formative-v10-e94027-s002.docx]

## Supplementary File 2: Baseline measures and scoring procedures

Baseline data included demographic information comprising age, gender, level of education, country of birth and relationship status. Baseline loneliness was ascertained using the UCLA 3-item Loneliness Scale (UCLA-3)[1], where a loneliness score (total score range 3–9) was calculated by summing the responses to the three items. A binary variable was generated: not lonely (scores 3–5) and lonely (scores 6–9) [2]

Social isolation was measured using the Lubben Social Network Scale-6 (LSNS-6), a 6-item instrument validated in older adults to measure social contacts and perceived social support [3]. Items are scored on a 6-point scale (0-5), with items summed up to result in total scores ranging from 0-30. A score of 12 or below indicates risk of social isolation.

Health-related quality of life was measured using the EQ-5D-5L [4], comprising five domains (mobility, self-care, usual activities, pain/discomfort, and anxiety/depression). Respondents rated each domain from 1 (no problems) to 5 (extreme problems). For reporting, responses were recoded to binary indicators to distinguish between presence versus absence of problems within each domain (1 = no problems; 0 = any level of problems), with each domain reported separately).

Mental wellbeing was measured via the Short Warwick-Edinburgh Mental Well-being Scale (SWEMWBS) [5], a 7-item scale capturing mental wellbeing over the past two weeks. Items are rated on a 5-point Likert scale (1–5) (1 = none of the time, 5 = all of the time), with scores summed and transformed into metric scores and three levels of wellbeing (low, moderate, high) using the SWEMWBS conversion table.

Extraversion was captured using two items from the Ten-Item Personality Inventory (TIPI) [6], (I see myself as (1) extraverted, enthusiastic and (2) reserved, quiet). Items are rated on a 7-point Likert scale (1 = strongly disagree to 7 = strongly agree). The second item was reverse-coded, and item scores were averaged. Scores were dichotomized at the sample midpoint (>4 vs ≤4) to indicate relatively higher versus lower extraversion.

Health and support information was also collected, including presence of sensory impairment (hearing, vision: yes/no); self-reported mental health conditions (yes/no); support from informal carer (yes/no); current/recent health and support services; and receipt of financial support (type).

### References

1.	Hughes ME, Waite LJ, Hawkley LC, Cacioppo JT. A Short Scale for Measuring Loneliness in Large Surveys:Results From Two Population-Based Studies. Research on Aging. 2004;26(6):655-72. doi: 10.1177/0164027504268574.

2.	Steptoe A, Shankar A, Demakakos P, Wardle J. Social isolation, loneliness, and all-cause mortality in older men and women. Proceedings of the National Academy of Sciences. 2013;110(15):5797-801. doi: 10.1073/pnas.1219686110.

3.	Lubben J, Blozik E, Gillmann G, Iliffe S, von Renteln Kruse W, Beck JC, Stuck AE. Performance of an abbreviated version of the Lubben Social Network Scale among three European community-dwelling older adult populations. Gerontologist. 2006;46(4):503-13. doi: 10.1093/geront/46.4.503.

4.	Herdman M, Gudex C, Lloyd A, Janssen M, Kind P, Parkin D, et al. Development and preliminary testing of the new five-level version of EQ-5D (EQ-5D-5L). Qual Life Res. 2011;20:1727-36. doi: 10.1007/s11136-011-9903-x.

5.	Stewart-Brown S, Tennant A, Tennant R, Platt S, Parkinson J, Weich S. Internal construct validity of the Warwick-Edinburgh Mental Well-being Scale (WEMWBS): a Rasch analysis using data from the Scottish Health Education Population Survey. Health Qual Life Outcomes. 2009;7(1):15. doi: 10.1186/1477-7525-7-15.

6.	Gosling SD, Rentfrow PJ, Swann WB. A very brief measure of the Big-Five personality domains. J Res Pers. 2003;37(6):504-28. doi: 10.1016/S0092-6566(03)00046-1.
