# Supplementary material for: Feasibility of Using a Wearable Device to Identify Loneliness Among Older Adults Living Alone: Mixed Methods Study
Source: JMIR Form Res. 2026 Oct 15;10:e94027. doi: 10.2196/94027 (PMC13671184; doi:10.2196/94027)
Supplement: Supplementary file 3 [file formative-v10-e94027-s003.docx]

## Supplementary File 3: Full recommendations for improvements on the device, instructions for user guide, and the activity log from phase 1 feedback and testing with older community members.

| Category of recommendations | Recommendations |
| --- | --- |
| The Device | - Too fiddly to plug the charger into the device – needs to be smaller/tighter hole, or closer to the outside - Concerned about it getting rained on; hitting or dropping it; spilling things on it while eating; wearing it around pets who might chew/play with it - Falls off, slippery -even with the clip– needs to be better secured;* - Lanyards – could be personalized/tailored to individual. Good for those with dexterity issues. May need choice* - Lanyards need to be adjustable* - Has to be long enough to lift over head – closure of lanyard too tricky - Collar of the lanyard did get a bit itchy – made more comfortable by putting over a jumper collar - Wearing it while also wearing a seatbelt is uncomfortable - Blue light very bright when charging at night - Knowing which way is on and off -?similar markers to charging, or write on/off on the device; to be more obvious/colored * - Need a way to know if the device is on and working (a light?) |
| Instructions | - Simple instruction on how it works and why to be able to explain to others who ask * - Advice needed on where it optimally needs to be worn – under/over clothing and location on torso;* - Don’t use front and back – incorporate with symbols etc. on device * - Strategies to remember to wear - put it near something you do in the morning so you don’t forget * - Reframe the troubleshooting as “we don’t anticipate anything going wrong, but here are a few things to consider” * - Make sure verbal instructions are clear as well – someone clear to explain; provide a follow up phone call the next day to go through again/answer any questions * - Perhaps put the key usable information i.e. on/off up front, index * |
| Activity Log | - Recommend filling it out in chunks i.e. morning and afternoon, then evening when wake up next morning; or as you go/after talking to people - How much of the time is ‘a little’, ‘some’ when feeling lonely? - Make the date larger/more obvious * - Provide pen* - Go through the activity log with participants to see if there’s anything you can’t read* - Provide options for bigger/smaller log size depending on participant - some may wish to type, print and stick* - Spiral bound so it can sit open; date and number the pages* - Include what type of relationship the person has with the person engaging in conversation – stranger, acquaintance, friend, child; * - Suggest to include the length & “quality” of chat/visit.* |
| * indicates where the research team were able to make modifications or provide alternatives following testing or feedback from older community members | |
