# Supplementary material for: Feasibility of Using a Wearable Device to Identify Loneliness Among Older Adults Living Alone: Mixed Methods Study
Source: JMIR Form Res. 2026 Oct 15;10:e94027. doi: 10.2196/94027 (PMC13671184; doi:10.2196/94027)
Supplement: Supplementary file 4 [file formative-v10-e94027-s004.docx]

| Guidelines | Location |
| --- | --- |
| 1. Describe the justification for using a mixed methods approach to the research question | Methods – Study design |
| 2. Describe the design in terms of the purpose, priority and sequence of  methods | Methods - Study design, Procedures |
| 3. Describe each method in terms of sampling, data collection and analysis | Methods - Participants; Procedures; Quantitative Measures; Qualitative Measures; Data Analysis (Phase 1 and Phase 2) |
| 4. Describe where integration has occurred, how it has occurred and who has  participated in it | Methods - Integration |
| 5. Describe any limitation of one method associated with the presence of the other method | Discussion - Data Capture Feasibility and Synchronization, Procedural and Implementation Limitations; Conceptual Limitations |
| 6. Describe any insights gained from mixing or integrating methods | Results – Integration, opening Discussion paragraphs |

Supplementary File 1: Good Reporting of a Mixed Methods Study (GRAMMS) checklist

O'Cathain, A., Murphy, E., & Nicholl, J. (2008). The quality of mixed methods studies in health services research. *Journal of health services research & policy*, *13*(2), 92–98. https://doi.org/10.1258/jhsrp.2007.007074
