# Supplementary material for: Feasibility of Using a Wearable Device to Identify Loneliness Among Older Adults Living Alone: Mixed Methods Study
Source: JMIR Form Res. 2026 Oct 15;10:e94027. doi: 10.2196/94027 (PMC13671184; doi:10.2196/94027)
Supplement: Supplementary file 5 [file formative-v10-e94027-s001.docx]

## Supplementary File 1: Activity and conversation log

 
